# Supplementary material for: Vastly different energy landscapes of the membrane insertions of monomeric gasdermin D and A3
Source: Commun Chem. 2025 Feb 6;8:38. doi: 10.1038/s42004-024-01400-2 (PMC11802827; doi:10.1038/s42004-024-01400-2)
Supplement: Supplementary file 2 — Supplementary Information [file 42004_2024_1400_MOESM2_ESM.pdf]

Supplementary Information - Vastly different energy landscapes of the membrane insertions of monomeric gasdermin D and A3

Viktoria Korn and Kristyna Pluhackova*

Stuttgart Center for Simulation Science, Cluster of Excellence EXC 2075

University of Stuttgart, Universitätsstr. 32, 70569 Stuttgart, Germany

E-mail: kristyna.pluhackova@simtech.uni-stuttgart.de

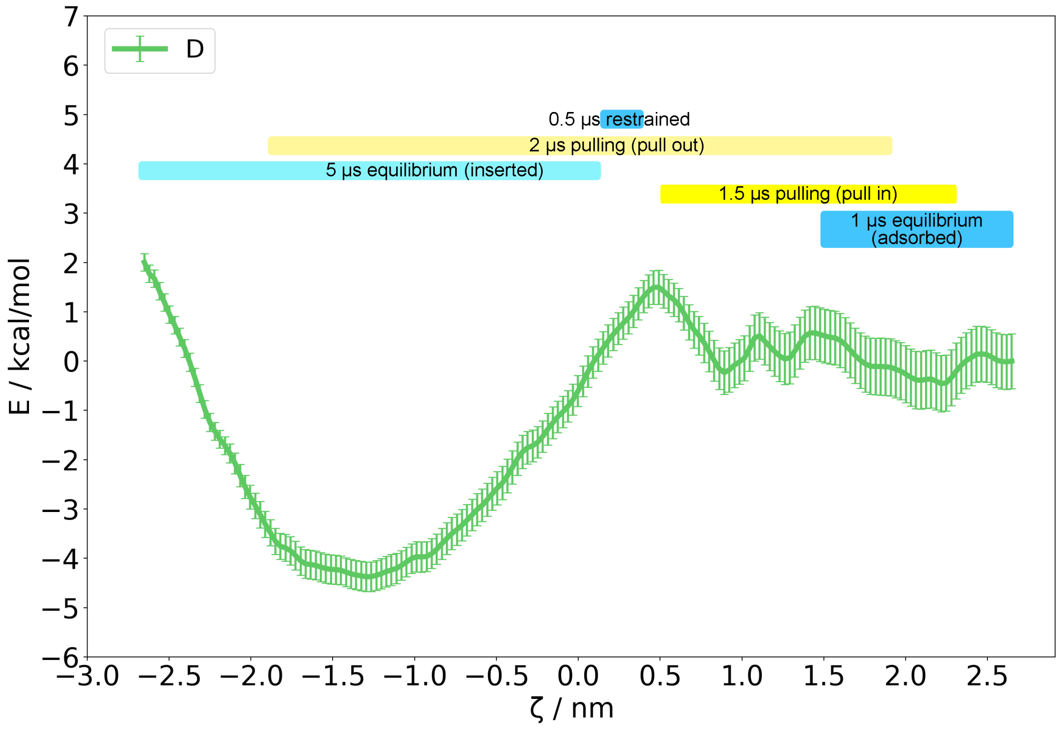

Figure S 1: Origin of the starting geometries for the geometric perturbation ('umbrella') simulations of GSDMD in an E. coli membrane (see main text Table 1).

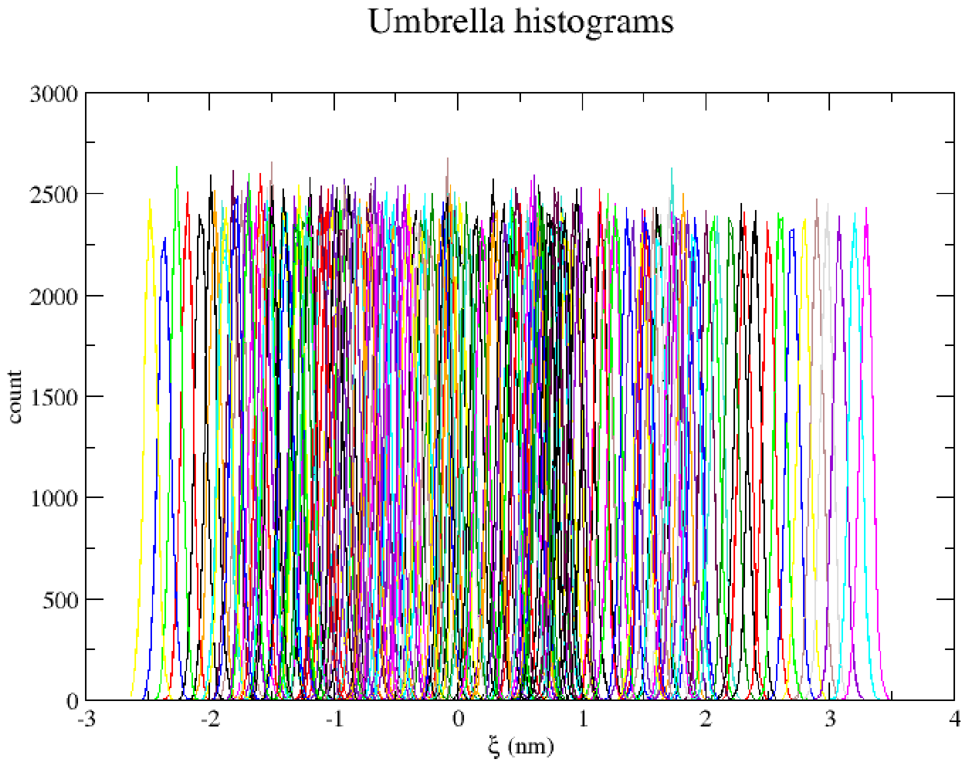

Figure S 2: Histograms of the 207 umbrella simulations used to construct the PMF of membrane insertion/excision of GSDMD into/from E. coli membrane. 52 of 91 snapshots taken from the pull-out simulation and 41 out of 42 snapshots taken from the pul-in simulation were equilibrated for 10 ns before performing the geometric perturbation ('umbrella') simulations. All others were used as-is for the geometric perturbation simulations to cover well the reaction coordinate.

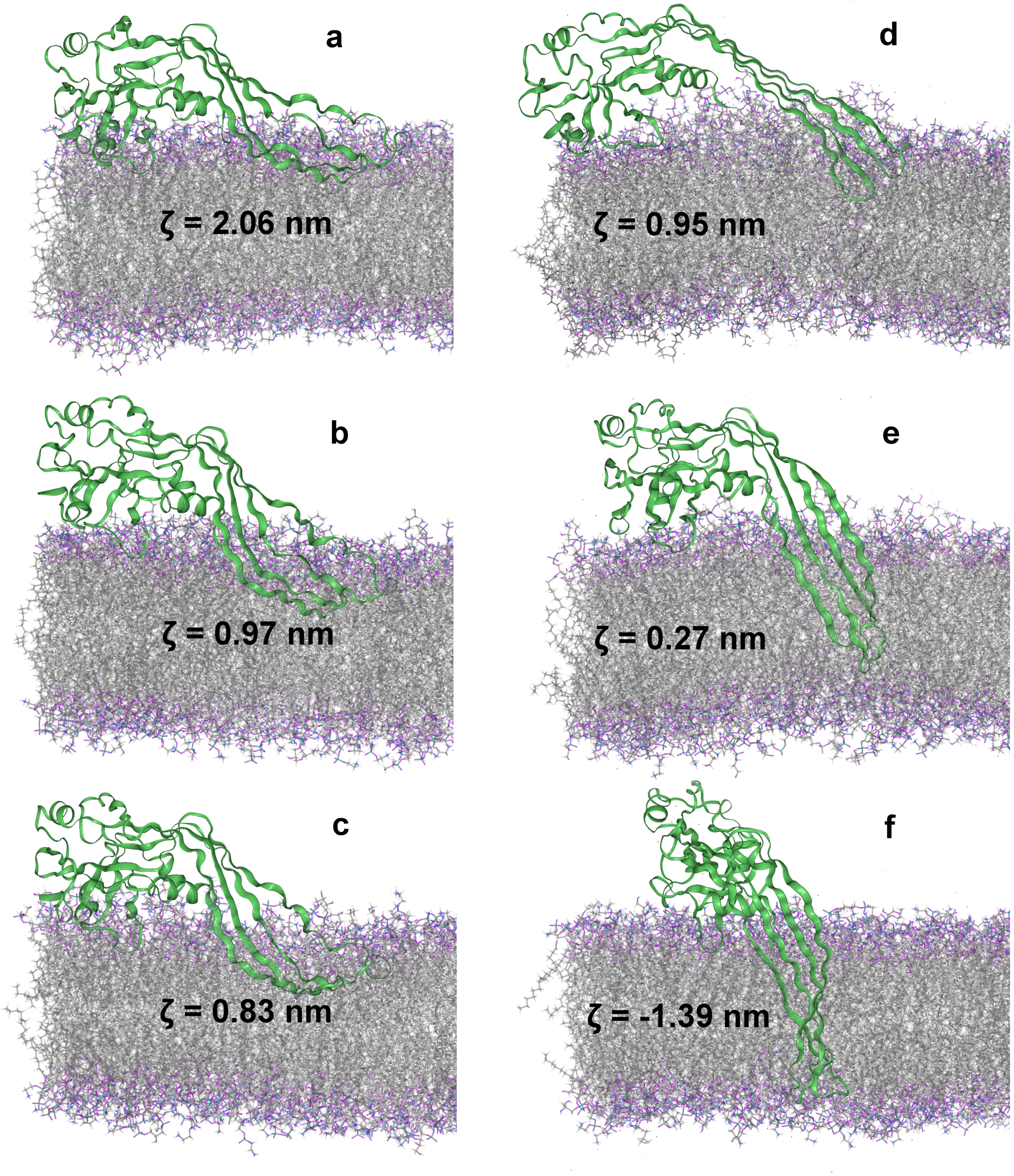

Figure S 3: Exemplary snapshots from the 'pull in' (a-c) and 'pull out' (d-f) pulling simulations which were used to extract starting structures for the geometric perturbation ('umbrella') simulations of GSDMD in E. coli membrane.

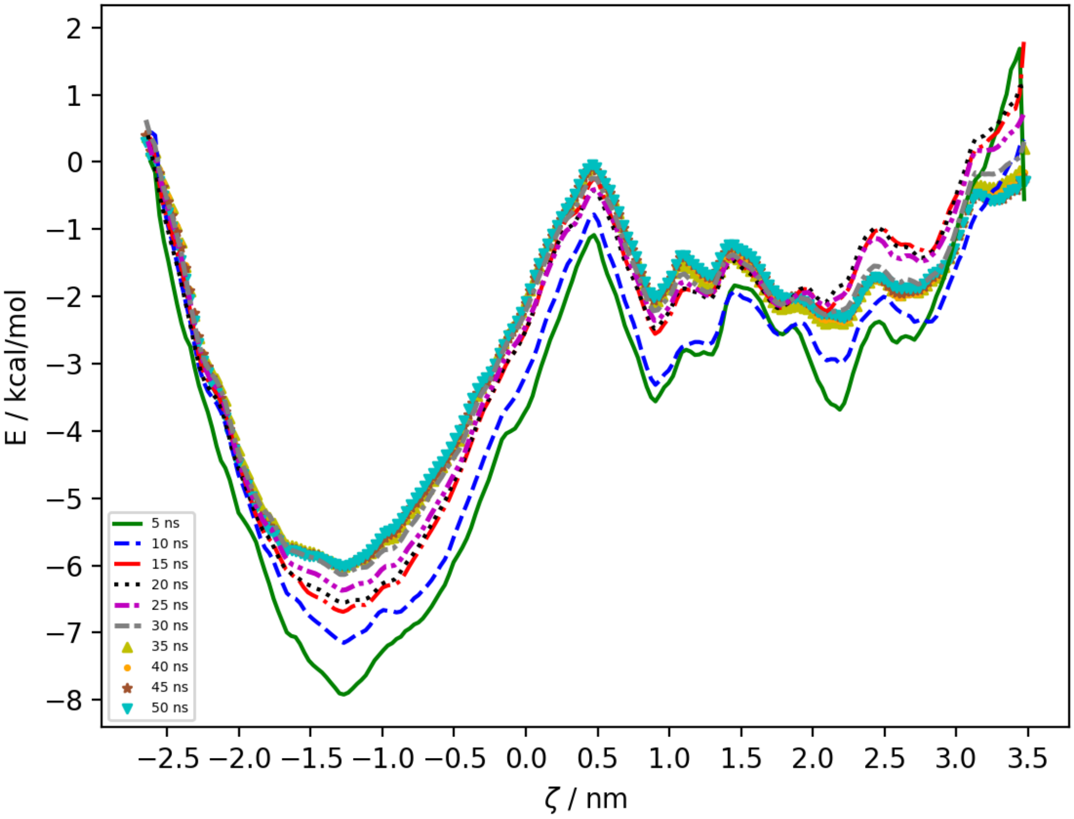

Figure S 4: Convergence of the PMF of membrane insertion/excision of GSDMD into/from E. coli membrane estimated by WHAM[1] from the time 0 and the indicated times. The overlap of the lines from 0-35 ns till 0-50 ns shows that the convergence was reached after 35 ns simulation time.

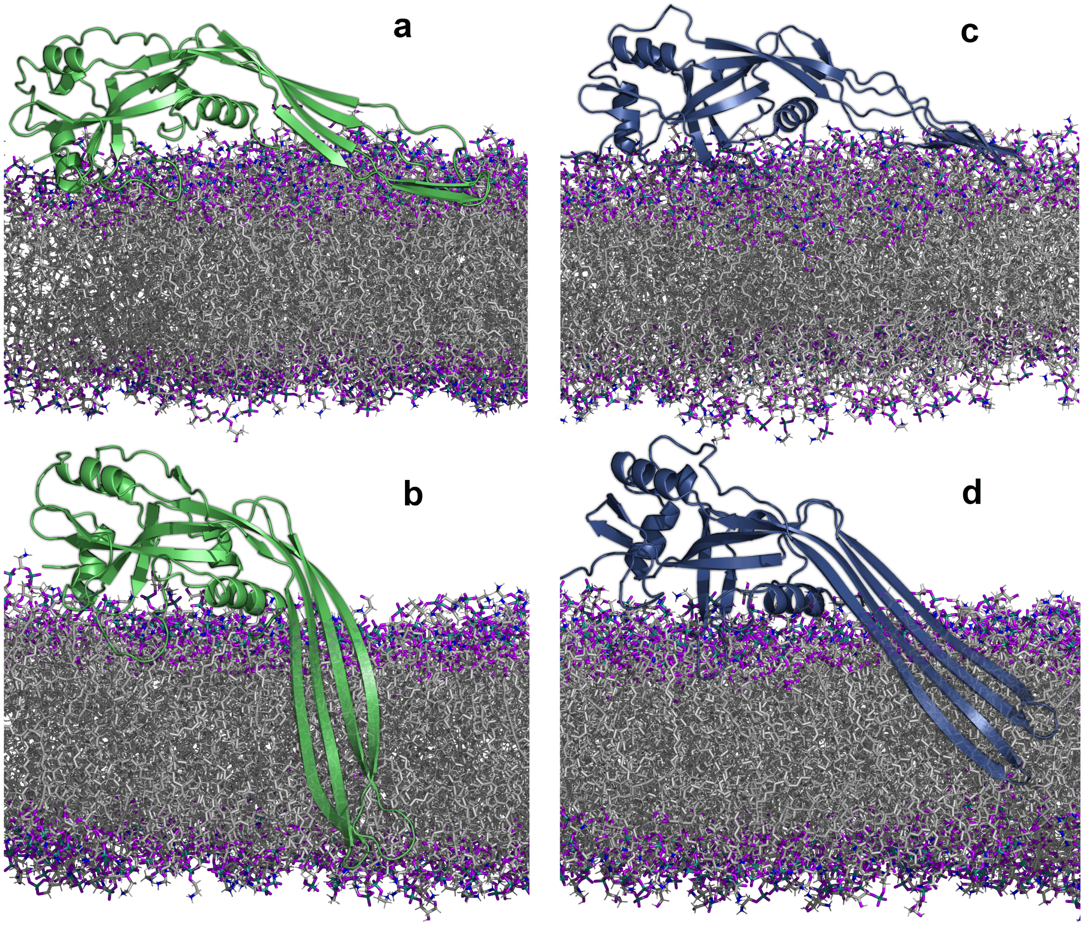

Figure S 5: Snapshots from the equilibrium simulations of GSDMD (a-b) and GSDMA3 (c-d). In both the adsorbed (a,c) and inserted states (b,d), GSDMD and GSDMA3 monomers assume different conformations due to the distinct chemical interactions based on the individual amino acid sequences of the two proteins. The proteins are shown as green (GSDMD) or navy (GSDMA3) cartoon and the E. coli membrane as grey-purple licorice.

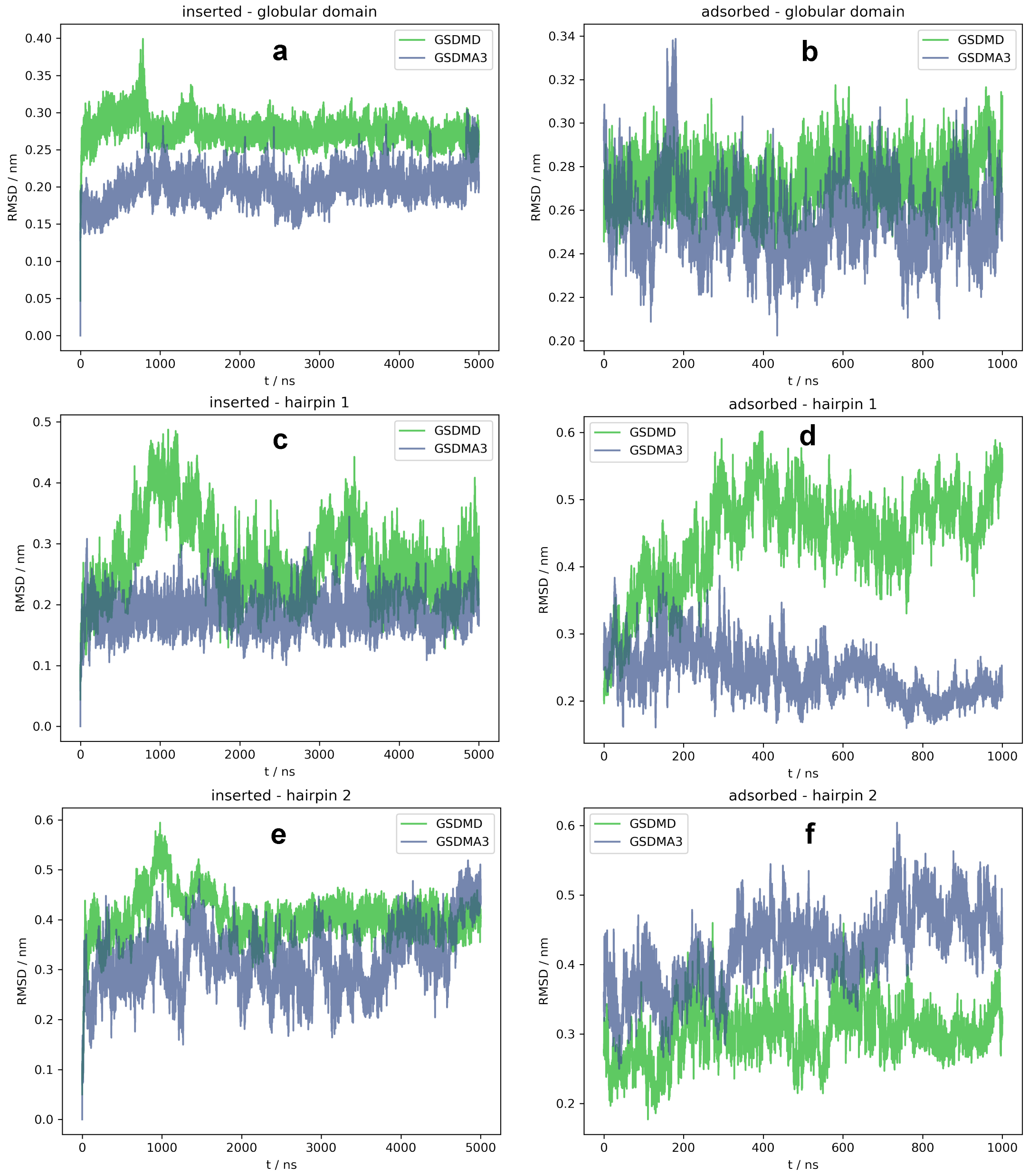

Figure S 6: RMSD over the equilibrium simulations of GSDMD (green) and A3 (navy) in the inserted (left column) and in the adsorbed state (right column). Only the backbone was used in the computation. The cryoTEM pore conformations were used as reference structures. In the inserted state, the globular domain (D: residues 1-86, 114-174, 205-241, A3: residues 1-85, 109-168, 195-237) of GSDMD is slightly more different from the pore conformation than that of GSDMA3 (a). The same applies to the β-hairpin one (D: residues 87-113, A3: residues 86-108) (c) and two (D: residues 175-204, A3: residues 169-194) (e). Compared to the globular domains, the β-hairpins are more flexible, except GSDMA3's hairpin one which is also quite rigid. In the adsorbed state, the globular domains are more flexible (b). In the adsorbed state the hairpins show a vastly different behavior for the investigated GSDMS: D's hairpin one is much more flexible than A3's hairpin one (d), while A3 possesses the more flexible hairpin two (f).

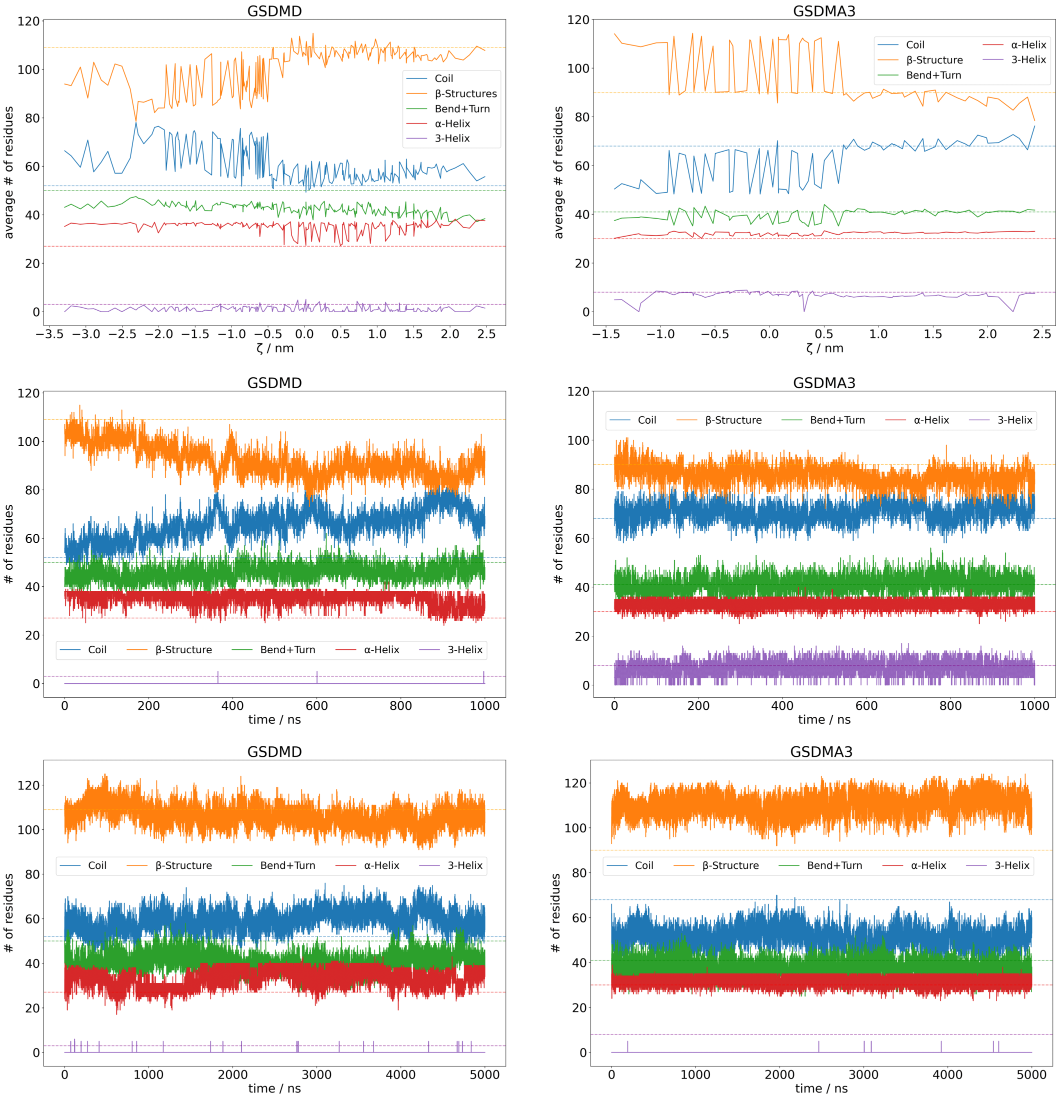

Figure S 7: DSSP secondary structure content analysis of GSDMD and GSDMA3 over the geometric perturbation simulations (top), the adsorbed 1 µs long (middle) and the inserted 5 µs long (bottom) simulations. For comparison, the secondary structure of the respective cryoTEM crystal structures is plotted as dashed lines. GSDMD (top left) unfolds some of its β-sheet structures during insertion, while GSDMA3 (top right) gains more β-structures, the deeper it is inserted. Both proteins have more β-sheets in the inserted equilibrium simulations (bottom) than in the adsorbed ones (middle).

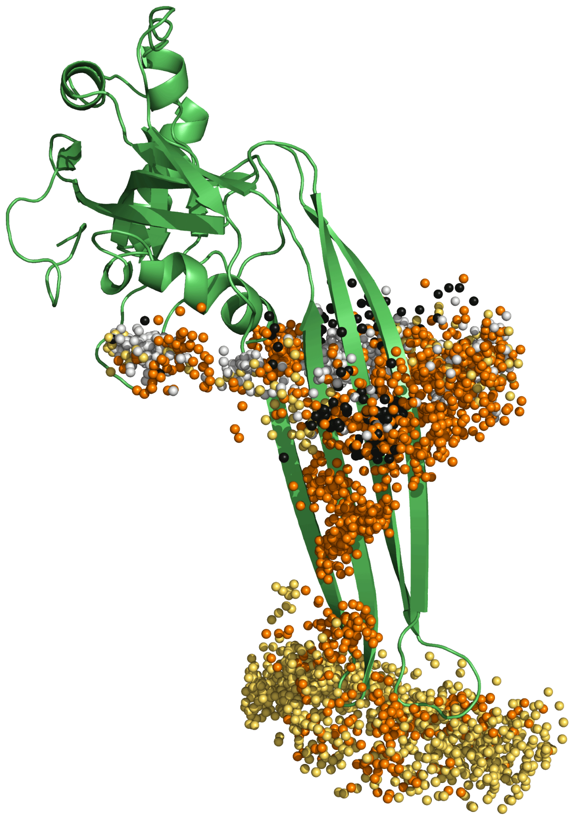

Figure S 8: The phosphorus atoms within 1 nm of the β-hairpins of GSDMD in the last 1 μs of the 5 μs-long simulation in a plasma membrane model by Schaefer et al.[2] GSDMD (conformation at 4.5 μs) is shown as green cartoon, the phosphorus atoms are visualized as spheres and colored according to the lipid type: Phosphatidylethanolamines are colored orange, phosphatidylinositols are colored black, phosphatidylserines are colored light grey, and phosphatidylcholines and sphingosylphosphatidylcholines yellow.

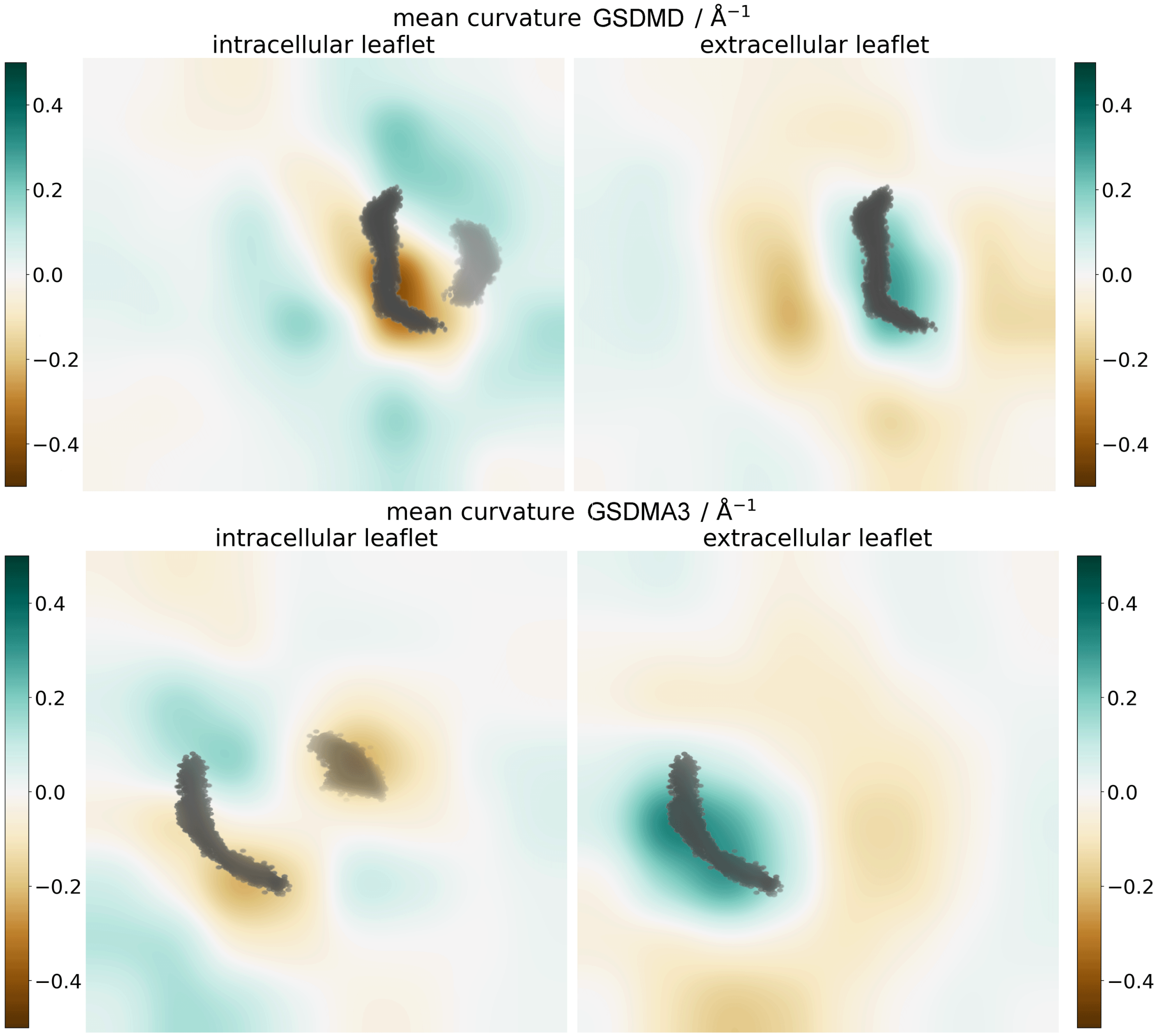

Figure S 9: Mean curvature over 5 µs equilibrium simulations of GSDMD (top) and GSDMA3 (bottom) inserted in E. coli membrane. The position of the center of geometry of the β-hairpins (dark grey) and the center of geometry of the globular domain (light grey) over the simulations are depicted as dots. Observed with the globular domain of gasdermins above the lipid bilayer, positive curvature is defined as a bump pointing upwards, while negative curvature describes a dip. The inserted proteins cause slight membrane deformations in their vincinity. For GSDMD, a stronger curvature at the inserted β-hairpins is visible (top left), corresponding to the higher amount of lipid headgroups (Main Figure 2) inside the membrane.

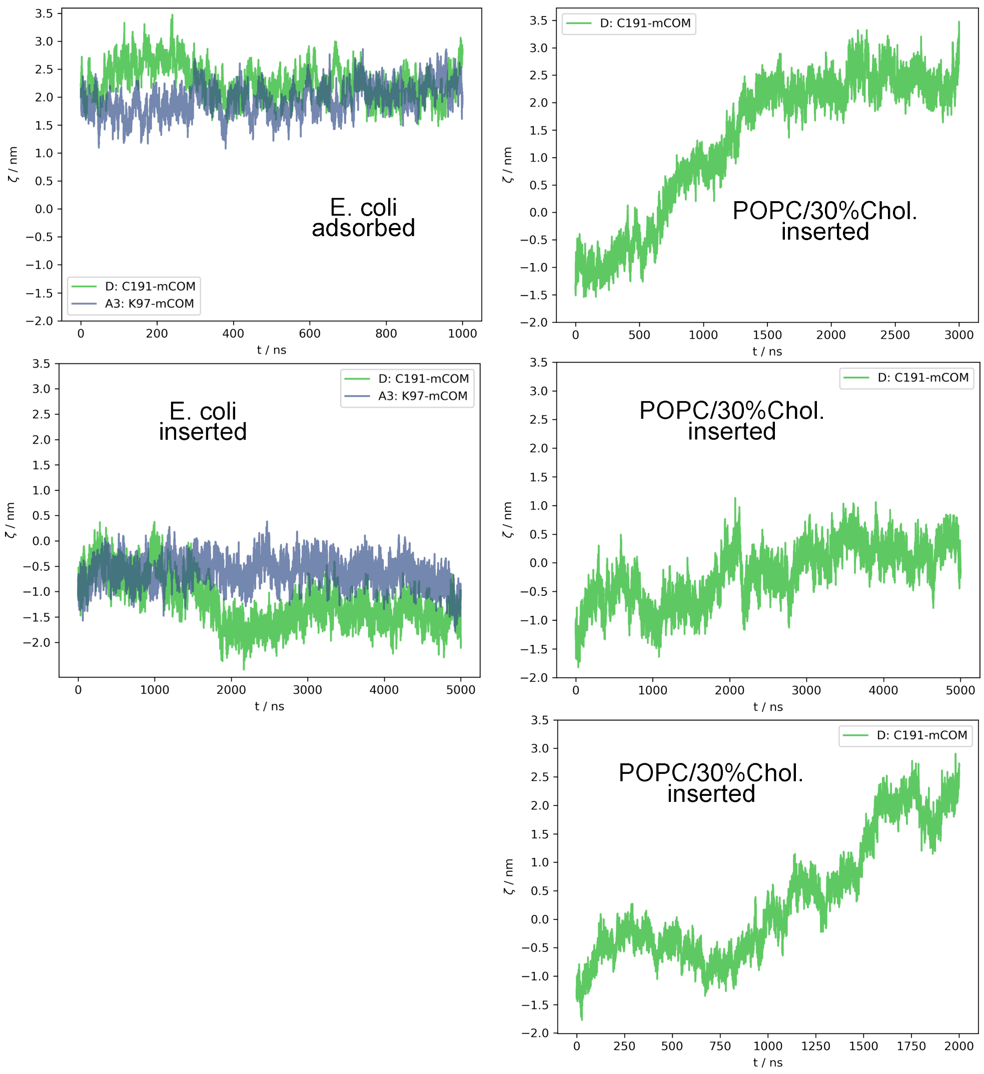

Figure S 10: Distance between the C_α of C191 (GSDMD) and K97 (GSDMA3) and the center of mass of the E. coli membrane along the membrane normal (mCOM) during the equilibrium simulations in the adsorbed state (top left) and the inserted state (middle left). In the right column, the distance between the C_α of C191 and the center of mass of the POPC/30%Cholesterol membrane along the membrane normal (mCOM) during three equilibrium simulations initiated in the inserted state is plotted.

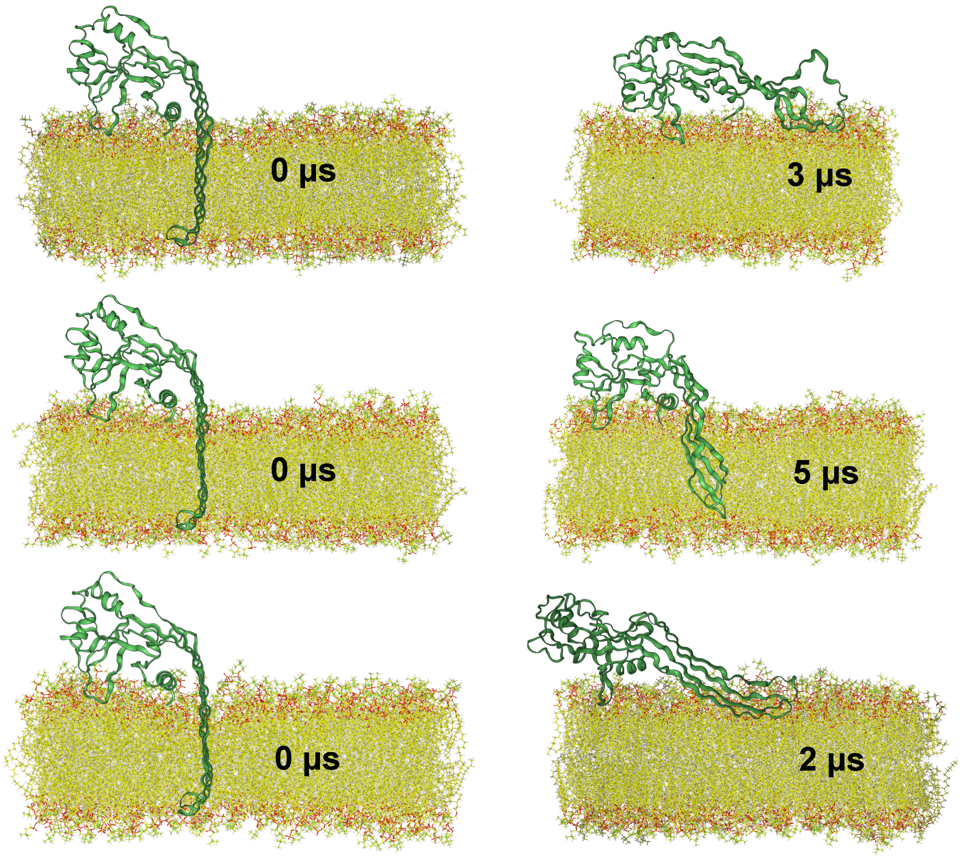

Figure S 11: Snapshots from the beginning (0 μs) and the end of three equilibrium simulations of GSDMD (shown as green cartoon) in a POPC/30%Cholesterol membrane (shown as yellow/red licorice). The membrane looks smaller in the right column due to the different rotation of the rectangular simulation box.

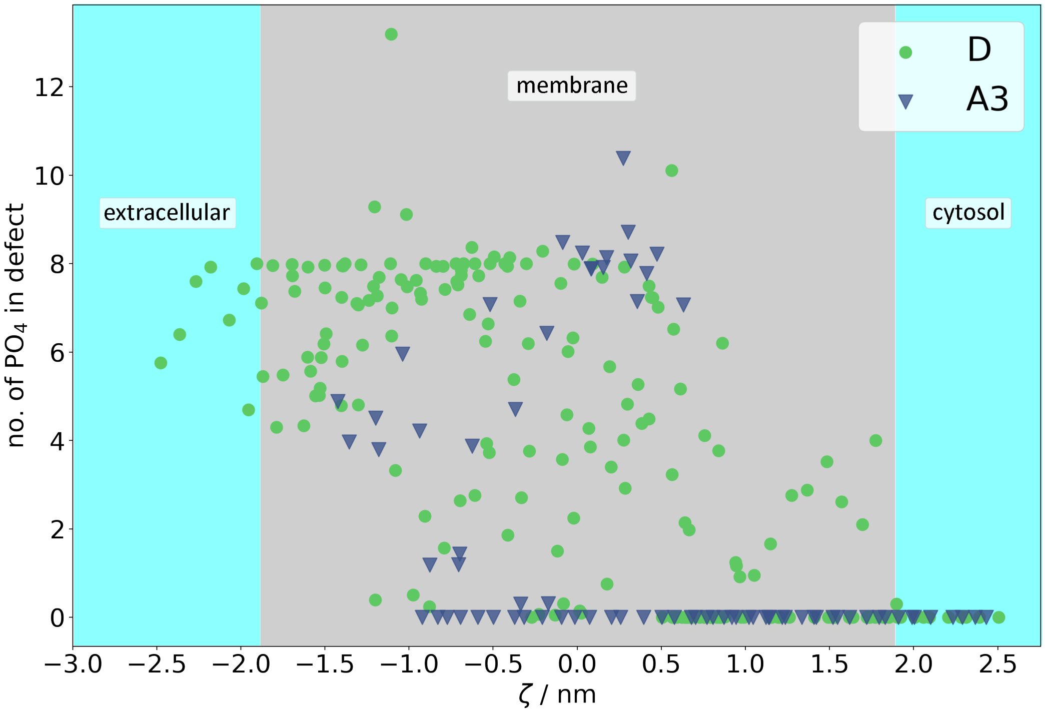

Figure S 12: Average number of phosphate headgroups in the membrane in the individual geometric perturbation ('umbrella') simulations of GSDMD (green) and GSDMA3 (navy) in E. coli membrane. The reaction coordinate ζ is defined as the distance between the C_α (C191 (GSDMD), K97 (GSDMA3)) and the center of mass of the membrane along the membrane normal.

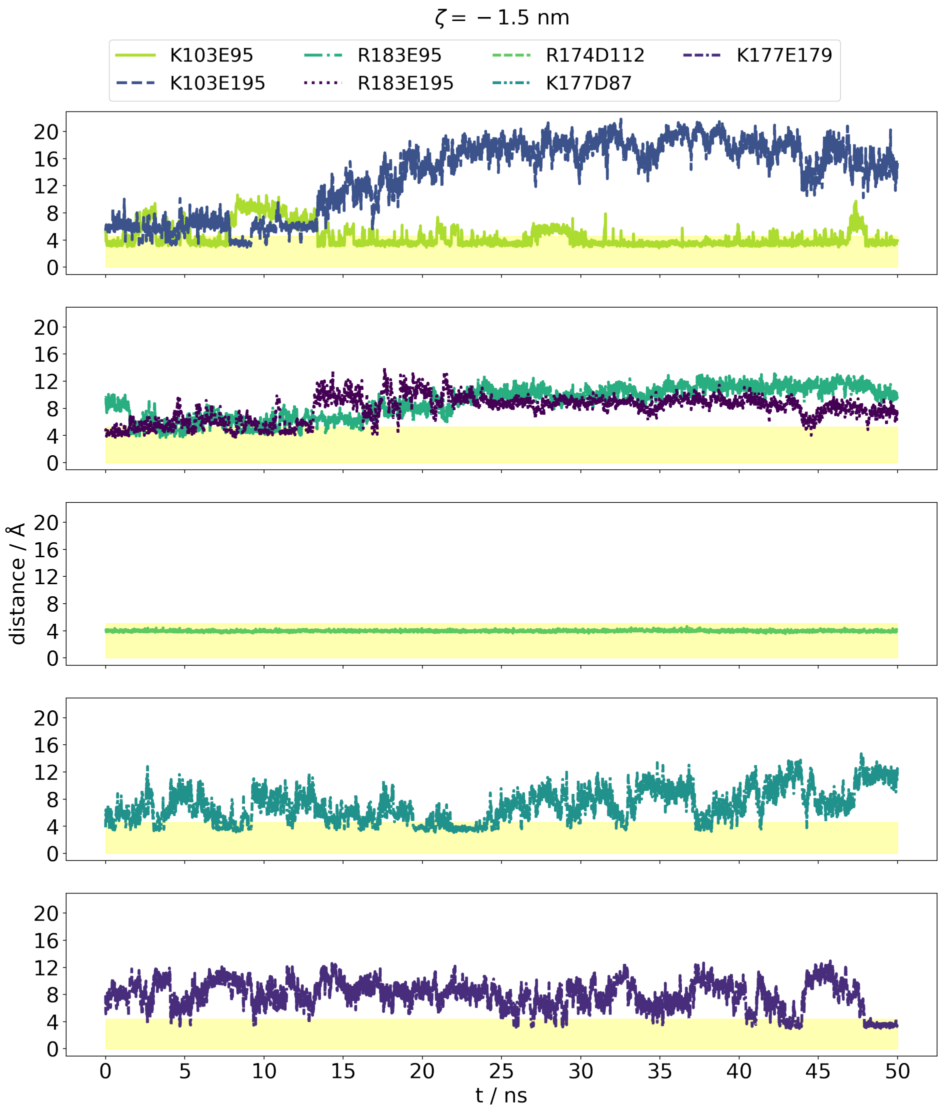

Figure S 13: Distances between the central atoms of the charged groups of lysine (K), arginine (R), glutamic acid (E), and aspartic acid (D) over the course of geometric perturbation ("umbrella") simulations. The light yellow rectangles mark the distances within which a salt bridge is formed. R174D112 is the most rigid salt bridge followed by K103E95. K177D87 and K177E179 show similar fluctuation behavior, while K103E195, R183E95, and R183E195 are more inconsistent. Typically the maximum salt bridge distance (N^+ - O^-) is defined as 4 Å. Here, in order to speed up the distance calculation, the atom selection was reduced to one central atom of each charged amino acid type and the salt bridge distance criteria were adapted accordingly: For lysine, the N atom in the terminal NH_3^+ group was selected, for glutamic acid the C atom of the terminal carboxyl group, for arginine the C atom of the guanidinium group, and for aspartic acid the C atom of the terminal carboxyl group. In the following, the maximum salt bridge distances were defined as 4.58 Å for lysine-glutamic acid, 5.28 Å for arginine-glutamic acid, 5.10 Å for arginine-aspartic acid, and 4.40 Å for lysine-aspartic acid, all based on the trigonometric configurations of the terminal groups.

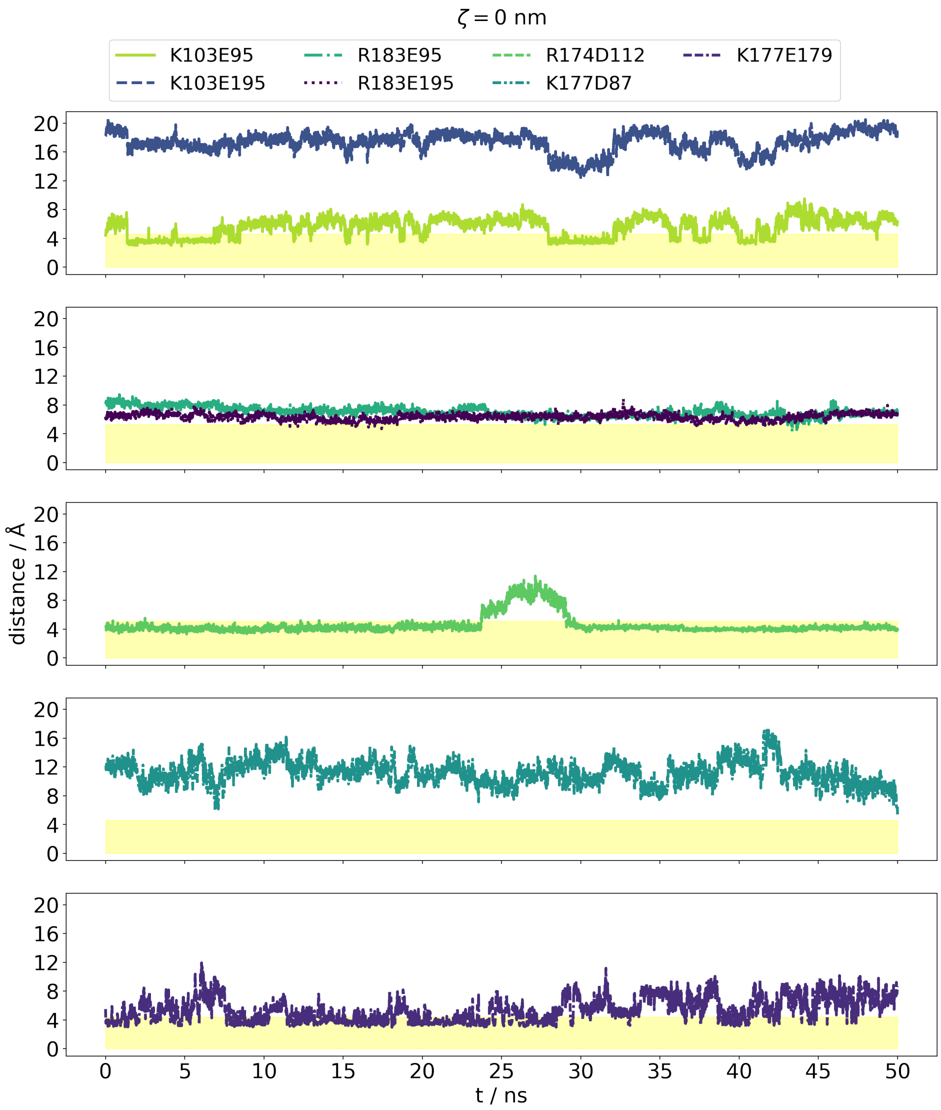

Figure S 14: Distances between the central atoms of the charged groups of lysine (K), arginine (R), glutamic acid (E), and aspartic acid (D) over the course of geometric perturbation ("umbrella") simulations. The light yellow rectangles mark the distances within which a salt bridge is formed. R174D112 forms the most stable salt bridge. K103E195 and K177D87 do not form one at $\zeta = 0$ nm. R183E95 and R183E195 stay at a stable distance not far from the salt bridge distance (light yellow). K103E95 and K177E179 fluctuate.

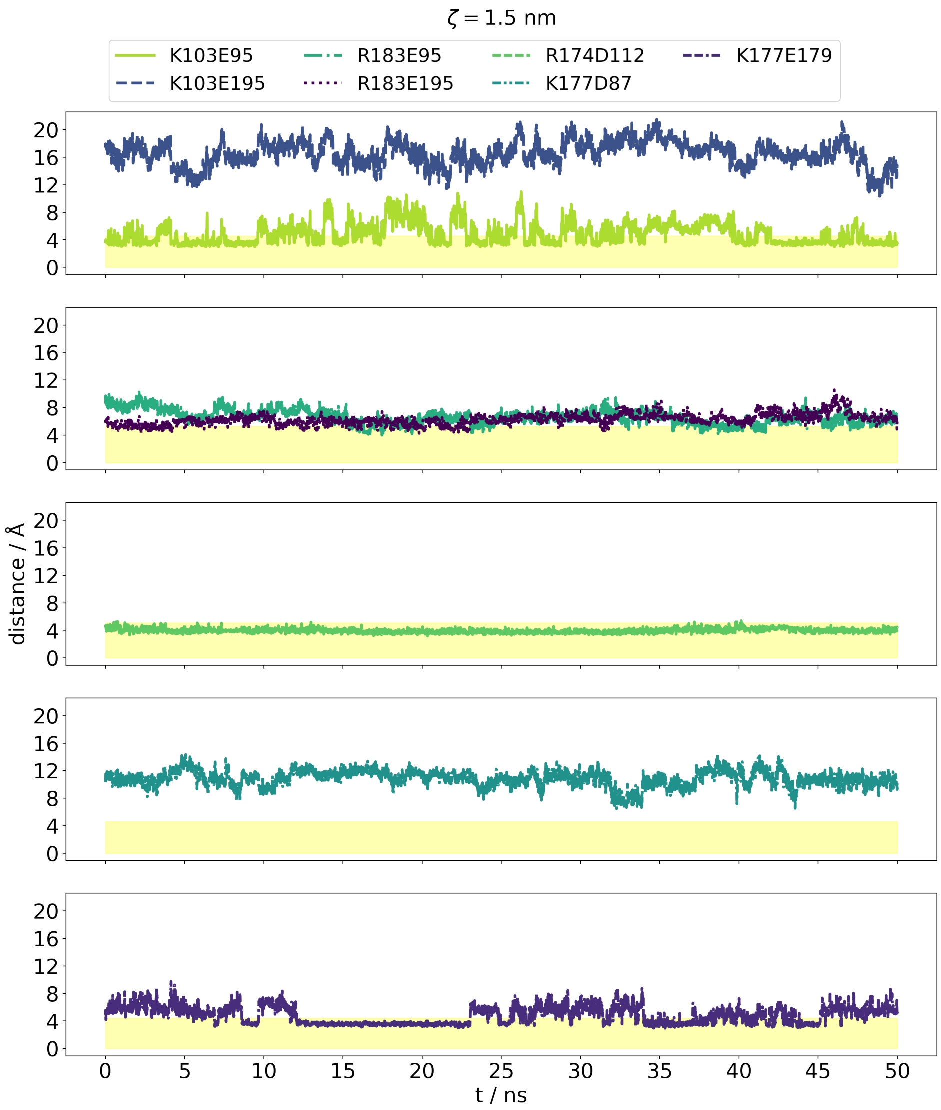

Figure S 15: Distances between the central atoms of the charged groups of lysine (K), arginine (R), glutamic acid (E), and aspartic acid (D) over the course of geometric perturbation ("umbrella") simulations. The light yellow rectangles mark the distances within which a salt bridge is formed. Deep inside the membrane, R174D112, too forms the most rigid salt bridge. Here, K177E179 forms a salt bridge most of the time, too. K103E195 and K177D87 do not form salt bridges. R183E95 and R183E195 fluctuate slightly, forming a salt bridge regularly.

Supplementary References

(1) Hub, J. S.; De Groot, B. L.; Van Der Spoel, D. g_wham - A Free Weighted Histogram Analysis Implementation Including Robust Error and Autocorrelation Estimates. *Journal of chemical theory and computation* **2010**, *6*, 3713–3720.

(2) Schaefer, S. L.; Hummer, G. Sublytic gasdermin-D pores captured in atomistic molecular simulations. *Elife* **2022**, *11*, e81432.
