# Supplementary material for: Vastly different energy landscapes of the membrane insertions of monomeric gasdermin D and A3
Source: Commun Chem. 2025 Feb 6;8:38. doi: 10.1038/s42004-024-01400-2 (PMC11802827; doi:10.1038/s42004-024-01400-2)
Supplement: Supplementary file 3 — Description of Additional Supplementary Files [file 42004_2024_1400_MOESM3_ESM.pdf]

Description of Additional Supplementary Files

File name: Supplementary Data 1

Description: Collection of data points used for plots.
